# Role of Pancreatic Stellate Cell-Derived Exosomes in Pancreatic Cancer-Related Diabetes: A Novel Hypothesis

**DOI:** 10.3390/cancers13205224

**Published:** 2021-10-18

**Authors:** Chamini J. Perera, Marco Falasca, Suresh T. Chari, Jerry R. Greenfield, Zhihong Xu, Romano C. Pirola, Jeremy S. Wilson, Minoti V. Apte

**Affiliations:** 1 Pancreatic Research Group, South Western Sydney Clinical School, Faculty of Medicine and Health, UNSW Sydney, Sydney 2052, Australia; h.c.perera@unsw.edu.au (C.J.P.); Zhihong.xu@unsw.edu.au (Z.X.); r.pirola@unsw.edu.au (R.C.P.); js.wilson@unsw.edu.au (J.S.W.); 2 Ingham Institute for Applied Medical Research, Sydney 2170, Australia; 3 Metabolic Signalling Group, Curtin Health Innovation Research Institute, Curtin Medical School, Curtin University, Perth 6102, Australia; Marco.Falasca@curtin.edu.au; 4 M.D Anderson Cancer Centre, Department of Gastroenterology, Hepatology and Nutrition, University of Texas, Houston, TX 75083, USA; STChari@mdanderson.org; 5 St Vincent Clinical School, Faculty of Medicine and Health, UNSW Sydney, Sydney 2052, Australia; jerry.greenfield@unsw.edu.au; 6 Healthy Ageing, Garvan Institute of Medical Research, Darlinghurst 2830, Australia; 7 Department of Diabetes and Endocrinology, St Vincent’s Hospital, Darlinghurst 3065, Australia

**Keywords:** pancreatic cancer-related diabetes, pancreatic stellate cells, exosomes, insulin resistance, biomarkers

## Abstract

**Simple Summary:**

Pancreatic Ductal Adenocarcinoma (PDAC) is a rapidly fatal disease. Diabetes mellitus is a major association of PDAC and is both a cause as well as a consequence of cancer. Notably, at the time of diagnosis of PDAC, more than 80% of patients have abnormal fasting blood glucose levels. Even more intriguing is the observation that a third of patients reports being diagnosed with diabetes within 3 years prior to their cancer diagnosis. This new onset diabetes, also called pancreatic cancer-related diabetes (PCRD) may be a harbinger of asymptomatic PDAC. Elucidating the mechanisms mediating PCRD will enable the identification of biomarkers for early diagnosis and/or novel molecular pathways that can be therapeutically targeted to improve patient outcomes.

**Abstract:**

Pancreatic ductal adenocarcinoma (PDAC) is a devastating condition characterised by vague symptomatology and delayed diagnosis. About 30% of PDAC patients report a history of new onset diabetes, usually diagnosed within 3 years prior to the diagnosis of cancer. Thus, new onset diabetes, which is also known as pancreatic cancer-related diabetes (PCRD), could be a harbinger of PDAC. Diabetes is driven by progressive β cell loss/dysfunction and insulin resistance, two key features that are also found in PCRD. Experimental studies suggest that PDAC cell-derived exosomes carry factors that are detrimental to β cell function and insulin sensitivity. However, the role of stromal cells, particularly pancreatic stellate cells (PSCs), in the pathogenesis of PCRD is not known. PSCs are present around the earliest neoplastic lesions and around islets. Given that PSCs interact closely with cancer cells to drive cancer progression, it is possible that exosomal cargo from both cancer cells and PSCs plays a role in modulating β cell function and peripheral insulin resistance. Identification of such mediators may help elucidate the mechanisms of PCRD and aid early detection of PDAC. This paper discusses the concept of a novel role of PSCs in the pathogenesis of PCRD.

## 1. Introduction

One of the major risk factors for Pancreatic Ductal Adenocarcinoma (PDAC) is type 2 diabetes mellitus, with type 2 diabetes patients reported to have a two-fold increased risk for the development of PDAC compared to the general non-diabetic population [1,2,3,4]. However, a unique form of diabetes, herein referred to as pancreatic cancer-related diabetes (PCRD), may also be a consequence of PDAC (PCRD is also called pancreatic cancer-associated diabetes or pancreatic cancer-induced diabetes; we have opted to use the term PCRD through this manuscript). PCRD is a subtype of Type3c (pancreatogenic) diabetes, which, according to the recent American Diabetes Association classification, is preferably termed pancreoprivic diabetes, i.e., diabetes secondary to pancreatic diseases, such as acute and chronic pancreatitis, trauma or pancreatectomy, neoplasia, cystic fibrosis, haemochromatosis, fibrocalculous pancreatopathy, rare genetic disorders and idiopathic forms [5].

The concept of PCRD was first put forward in 2008, when Chari et al. [6] observed that 52% of patients newly diagnosed with PDAC had a history of new onset diabetes (diagnosed within the past 2–3 years prior the diagnosis of PDAC) [7,8], while 80% of patients had hyperglycaemia, as assessed by fasting glucose levels, at the time of diagnosis of PDAC. Clinical prediction models have also shown that there is an increased risk of developing PDAC in patients with new onset diabetes [9,10]. Furthermore, a population-based study reported that nearly 1% of diabetes patients would be diagnosed with PDAC within 3 years of the diagnosis of diabetes [11].

New onset diabetes in PDAC often differs from longstanding type 2 diabetes in significant ways, as shown in Table 1. Such patients:

(i) Do not consistently exhibit hyperinsulinaemia [12,13];
(ii) Paradoxically experience marked weight loss, which starts even before the development of diabetes [14]; and
(iii) Can experience amelioration of glucose intolerance, including resolution of diabetes, upon resection of the pancreatic tumour [15,16].

The above observations suggest that PCRD is a paraneoplastic phenomenon caused by a developing tumour and that new onset diabetes may be a harbinger of asymptomatic PDAC [8]. It is possible that the growth of cancer impairs islet cell function and/or predisposes to peripheral insulin resistance resulting in hyperglycaemia, hyperglucagonaemia and diabetes. Notably, hyperglycaemia is associated with poor overall survival in PDAC [17,18], indicating that a diabetic state may facilitate cancer progression.

The observation that a subset of PDAC patients has a history of new onset diabetes (i.e., a diagnosis of diabetes in the 2–3-year period *prior to* diagnosis of cancer) affords a window of opportunity for earlier diagnosis of PDAC. Understanding the molecular mechanisms underpinning the relationship between carcinogenesis and diabetes could help identify potential biomarkers of early PDAC.

The earliest pre-malignant changes of PDAC are known as pancreatic intraepithelial neoplasms (PanINs) [19]. It is now widely acknowledged that progression of PanINs to overt cancer is not only dependent on the behaviour of cancer cells themselves but is also critically influenced by the surrounding stroma/desmoplasia [20,21,22]. It is possible that cancer cells and/or the surrounding stromal cells, specifically PSCs (the key producers of the collagenous stroma of PDAC), secrete factor(s) that act via a ‘humoral’ pathway to (i) inhibit islet cell function at a distance from the PanINs or cancer foci and/or (ii) affect insulin signalling pathways leading to insulin resistance. These factor(s), if identified and characterised, could be used as (i) biomarkers, particularly in the case of new onset diabetes to identify patients with PDAC that is early or at a premalignant stage, and (ii) as potential therapeutic targets to prevent progression to overt PDAC.

A few recent studies have postulated the involvement of exosomes, i.e., small extracellular vesicles with size range of 40 to 160 nm [23,24], derived from PDAC cells in PCRD, supporting the idea of a paraneoplastic origin of PCRD [25,26,27]. However, there is a paucity of studies probing the plausible role of PSC-derived exosomes in PCRD. Thus, this concept paper aims to address the likely involvement of PSC and PDAC cell-derived exosomes (and their cargo) in PCRD. Characterisation of the functions of such exosomal cargo may provide valuable insights for early detection of PDAC, and consequently, improved patient outcomes.

## 2. What Is Known about Pancreatic Cancer-Related Diabetes?

The incidence of diabetes is substantially higher in PDAC compared to other common cancers. Aggarwal et al. [28] reported that nearly 70% of PDAC patients had diabetes, in contrast to lung cancer (19.6%), breast cancer (19.4%), prostate cancer (14.8%) and colorectal cancer (20.7%). Except for PDAC, the prevalence of diabetes in other cancers was similar to that of healthy controls (23.5%). As mentioned above, patients with PCRD often exhibit impaired glucose tolerance, hyperglycaemia or overt diabetes 2–3 years prior to the diagnosis of cancer [12,13]. Interestingly, this hyperglycaemic state is often ameliorated/reversed following surgical resection of the pancreatic tumour [14,29], a phenomenon that is counter to what one would expect after pancreatic resection in a patient with diabetes. In addition, PCRD is associated with significant weight loss, which is prominent even before the diagnosis of cancer, whereas longstanding diabetes is usually associated with weight gain and is better controlled by weight loss [30]. A retrospective study by Sah et al. [31] reported several metabolic (serum glucose, serum lipids and body temperature) and soft tissue changes (subcutaneous and visceral adipose tissue and muscle mass) in patients in the months prior to the diagnosis of PDAC, supporting the concept that such metabolic changes may foreshadow PDAC. Insulin resistance, which is notionally associated with obesity in diabetes, has also been reported in PCRD patients, even though the latter patients tend to be very lean [32]. The insulin resistance in PCRD patients is also known to be ameliorated following tumour resection [16].

Histologically, the pancreata of patients with PCRD exhibit a decrease in the size of islets as well as a reduction in the number of β cells [33]. Amyloid plaques in islets, a hallmark feature of diabetes [34] are characteristically absent or low [33] in the pancreata of patients with PCRD. It has also been reported that Connexin 26, a gap junction protein, is highly upregulated in islets of patients with PDAC with impaired glucose tolerance compared to healthy controls and patients with chronic pancreatitis [35]. Upregulation of vanin-1 and matrix metalloproteinase 9 genes, known to be involved in inflammation, have been postulated as a potential indicator of PCRD [36]. However, the reliability and accuracy of these ‘biomarkers’ for the diagnosis of PDAC in the setting of new onset diabetes remains unproven.

PDAC is typically diagnosed at an advanced, unresectable stage, making it difficult to offer any curative treatments. However, given that the prevalence of PDAC in the general population is much lower than many other cancers [28], population screening is not seen to be cost-effective or prudent. Nevertheless, there is general agreement in the field that the identification of a biomarker for early screening of a ‘high-risk’ group should remain a research priority. In view of its possible importance as a harbinger of PDAC, understanding the characteristics and mechanisms of PCRD would be of significant value for its early diagnosis [37] and may open up new avenues for its treatment.

## 3. Mechanisms of Type 2 Diabetes Mellitus

**Figure 1:**
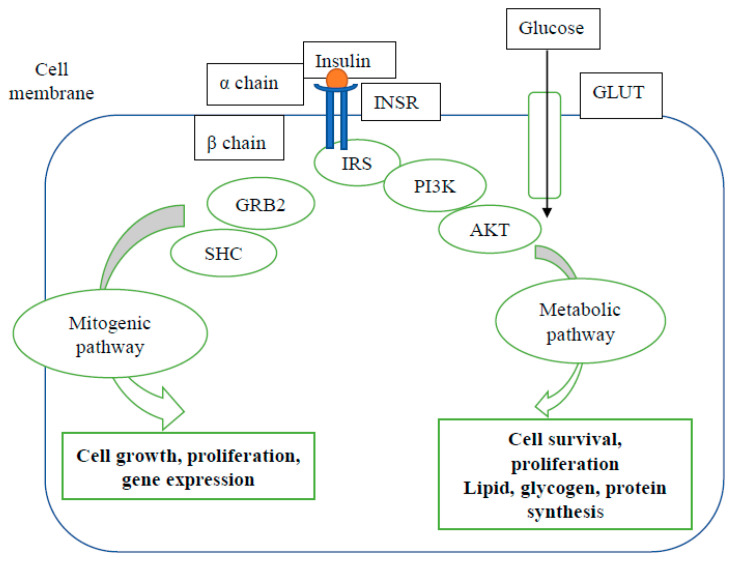
Insulin signalling pathway. Upon binding with insulin, the insulin receptor (INSR) autophosphorylates and recruits various substrates. The two major arms of insulin signalling are mitogenic (initiated by Growth factor receptor-bound protein 2 (GRB2) and Src homology and collagen (SHC)) and metabolic (initiated by insulin receptor substrate (IRS) proteins). In the metabolic pathway, phosphorylated IRS activates phosphoinositide-3-kinase (PI3K) with subsequent phosphorylation of protein kinase B (AKT). The glucose transporters (GLUTs) are the main insulin-responsive glucose carriers that transport glucose between the cell membrane and intracellular organelles. The mitogenic pathway regulates cell growth, proliferation and gene expression, while the metabolic pathway regulates cell survival, proliferation, lipid, glycogen and protein synthesis (figure adapted from Peterson et al. [39] and modified by the authors).

β-cell dysfunction and insulin resistance are the two known pathogenic mechanisms for the development of diabetes. Usually, disproportionate elevation in circulating proinsulin compared to insulin is deemed a marker of impaired β-cell function, while elevated circulating insulin plus proinsulin levels denote peripheral insulin resistance [38]. Insulin resistance is the reduced ability of target cells (hepatocytes, adipocytes and skeletal myocytes) to elicit a glucose lowering response to insulin. In health, insulin receptors (INSRs) on the target cell membranes bind to insulin, resulting in the activation of downstream signalling pathways that mediate glucose uptake and synthesis of major macromolecules, as depicted in Figure 1.

However, when the cells are no longer sensitive to normal plasma level of insulin and unable to elicit a glucose lowering response (involving cellular glucose uptake, glycogen synthesis, suppression of lipolysis and gluconeogenesis), they are considered to be insulin resistant [39,40,41]. Several factors, including (central) obesity, via the release of free fatty acids and cytokines/adipokines and, more controversially, chronic hyperinsulinaemia, have been implicated as potential triggers for insulin resistance in cells. Moreover, in vitro and in vivo studies have established that impaired insulin action in target cells occurs due to defects at the receptor level (decreased number of receptors at the cell surface) or post-receptor flaws in the downstream signal transduction pathways [39,42,43].

In type 2 diabetes, individuals develop peripheral insulin resistance prior to clinically evident diabetes, increasing the workload of β cells to increase insulin secretion, leading to a rise in fasting plasma insulin levels (hyperinsulinaemia). It is believed that elevated plasma glucose levels due to peripheral insulin resistance stimulate β cells to elicit a compensatory response to increase the levels of insulin. This is achieved via several adaptive mechanisms, such as increased β cell proliferation, upregulation of mitochondrial activity, stimulation of the unfolded protein response and augmented glucose metabolic pathways, leading to increased insulin production and secretion. Unfortunately, however, these adaptive mechanisms seem to be transient, since eventually, β cells lose the ability to meet the demands of prolonged glucose or lipid exposure. Studies have shown that β cell exhaustion and/or loss occurs due to several factors including progressive ER stress, mitochondrial dysfunction, oxidative stress, inflammation and accumulation of islet amyloid [39,44].

## 4. Possible Mechanisms of Pancreatic Cancer-Related Diabetes (PCRD)

As noted above, clinical and histological findings related to diabetes in PDAC are different from longstanding diabetes in several ways; however, the exact mechanisms of PCRD are yet to be elucidated. The limited research conducted in this area with in vitro and in vivo studies demonstrates both insulin resistance and β cell dysfunction in PDAC [8]. A study by Liu et al. [45] using skeletal muscle biopsies from PDAC patients with diabetes has shown impaired glycogen synthesis, but unaltered INSR, IRS and GLUT4 expression, suggesting post-receptor defects in the insulin signalling pathway in PCRD. Moreover, a decrease in insulin release has been noted in rat pancreatic islets incubated with conditioned media of the cancer cell lines, Panc-1 and HPAF cells or when co-cultured with Panc-1 and HPAF cells [46,47]. Another study revealed that glucose tolerance and insulin release was impaired in chemically-induced pancreatic cancer in Syrian hamsters [48].

Clinical studies have shown that the β cell response (as measured by response to oral glucose load, hyperglycaemic clamp or glucagon stimulation) is impaired in PDAC [16,49,50]. This observation is supported by a study of patients with PDAC by Chari and colleagues [12] employing Homeostasis Model Assessment (HOMA), in which they reported decreased β cell function, increased fasting glucose and modestly increased insulin resistance. In patients with PDAC and diabetes, increased serum glucagon, somatostatin and islet amyloid polypeptide (IAPP) levels were found to revert to normal following tumour resection, indicating an influence of tumour cells on the profiles of these hormones [29]. Permert et al. [32] demonstrated diminished glycogen synthesis in skeletal muscle cells incubated with tumour extracts from PDAC patients implicating a possible tumour-mediated effect. However, this early study failed to confirm the involvement of any of the endocrine factors measured in the tumour extracts, which is an important omission, since the observed effects could have been mediated by other factors secreted by the cells in the tumour microenvironment. This gap in knowledge and the limitations of earlier studies warrants further research on the involvement of surrounding stromal cells in the pathogenesis of PCRD.

## 5. Pancreatic Stellate Cells and Their Role in PDAC and Diabetes

Pancreatic stellate cells (PSCs), as the name implies, are star-like, resident cells in the pancreas accounting for 4–7% of pancreatic parenchymal cells [51]. PSCs express both mesenchymal and neuroectodermal markers, but are deemed to be of mesenchymal origin largely due to increased nestin expression [52]. A proportion of the PSC population is thought to be derived from bone marrow progenitors [53]. PSCs are now established as the predominant collagen-producing cells in the stroma of PDAC.

In their quiescent state in the healthy pancreas, PSCs store retinoids (vitamin A), largely as retinyl palmitate, in association with intracellular albumin [51]. However, the exact role of these stored retinoids remains to be fully elucidated. PSCs provide a supporting extracellular matrix (ECM) network for pancreatic lobules, acini, ducts and islets of Langerhans. Additionally, these cells secrete metalloproteinases (MMP-2, -9 and 13) as well as their inhibitors, and thus, they are involved in regular ECM turnover [54]. PSCs may also play an intermediary role in cholecystokinin-stimulated digestive enzyme secretion by acinar cells. These cells have been shown to express cholecystokinin receptors (CCK1 and 2) [55,56]. When exposed to CCK, PSCs synthesise and secrete the neurotransmitter acetylcholine [56], which in turn acts on muscarinic receptors on acinar cells to induce digestive enzyme secretion.

Inflammation or tumorigenesis can activate quiescent PSCs into a myofibroblast-like phenotype characterised by the loss of cytoplasmic vitamin A stores and increased expression of the activation marker α-SMA (alpha smooth muscle actin, a cytoskeletal protein). Activated PSCs can proliferate and migrate extensively and synthesise excessive amounts of extracellular matrix proteins [57,58,59]. Importantly, PSCs and PDAC cells have a reciprocal relationship that supports the survival of both cell types and also facilitates cancer progression [60]. For example, PSCs enhance cancer cell proliferation, decrease apoptosis, induce cancer cell migration in association with epithelial to mesenchymal transition (EMT) and enhance cancer cell stemness, thus promoting tumour growth, metastasis and recurrence [61,62,63,64]. In turn, cancer cells accelerate PSC proliferation, migration and ECM synthesis, thereby subverting PSCs to their own advantage [65,66]. These interactions between PSCs and cancer cells lead to a highly desmoplastic and hypoxic tumour micro-environment that can mediate resistance to chemo- and radiotherapy [67].

Tumour microenvironment consists of immune cells, endothelial cells, and cancer-associated fibroblasts (the latter predominantly comprising activated pancreatic stellate cells). It is now recognised that PSCs in pancreatic cancer exhibit significant inter- and intra-tumour heterogeneity. In this regard, the earliest report was by Ikenaga et al. [68], demonstrating two populations of PSCs in patients with pancreatic cancer—positive or negative for the membrane metalloproteinase CD10, with the former being associated with worse outcomes. Subsequently, Öhlund et al. [69] described different subtypes of cancer-associated fibroblasts (CAFs) in both mouse and human pancreatic cancer—high α-SMA expressing CAFs located adjacent to neoplastic cells (myofibroblastic CAFs; myCAFs) and low α-SMA-expressing cells at a distance from neoplastic cells, but with elevated IL-6 expression (inflammatory CAFs; iCAFs). Most recently, Neuzillet and colleagues have classified CAFs into four subtypes (A, B, C and D) based on transcriptomic analysis. These subtypes demonstrated unique molecular and functional features and showed different effects on prognosis. Furthermore, this study revealed that PSCs are closely related to subtypes B and C, which predominantly express myosin II and podoplanin, respectively [70].

**Figure 2:**
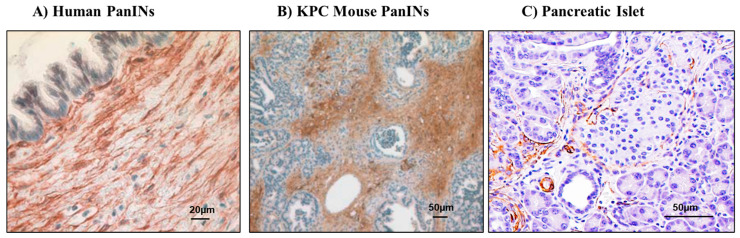
This figure depicts activated PSCs (α-SMA + ve brown stain) surrounding (**A**) PanINs in human pancreatic tissue, (**B**) PanINs in pancreas from a genetically modified mouse model (LSL-KrasG12D/ + Pdx-Cre mice) and (**C**) a pancreatic islet from (LSL-KrasG12D/ + Pdx-Cre mice). Images (**A**,**B**) are adapted from Wilson et al. [79], while (**C**) is the original photomicrograph from the authors’ work.

As noted earlier, diabetes associated with PDAC can occur even before the diagnosis of PDAC or at a very early stage of cancer [6,71]. Invasive PDAC arises from non-invasive pre-malignant lesions called PanINs. The timeline of development of PanINs to advanced cancer is variable but can be up to 10 years before the diagnosis of cancer [72,73,74]. Of note is the fact that activated pancreatic stellate cells are already present around these earliest lesions of PDAC (Figure 2). Some studies postulate that PSCs may act to restrain early-stage cancer growth [75,76], but it is now widely accepted that eventually cancer cells subvert PSC function to their own benefit, as discussed in recent reviews by Pothula et and [77] Mekapogu et al. [78]. However, the role of PSCs in pancreatic cancer-related diabetes has never been studied.

Although the first description of PSCs in 1998 [51] described their location as adjacent to the basolateral aspect of pancreatic acinar cells, more recently, PSCs have also been identified around pancreatic islets (termed islet stellate cells (ISCs)) [80]. Both human and rat ISCs have been successfully isolated, cultured and characterised and are assumed to mediate the islet fibrosis typical of type 2 diabetes [81,82], ISCs have been reported to be phenotypically akin to PSCs, but exhibit certain functional differences from ‘conventional’ PSCs. In addition, ISCs tend to activate more rapidly but show slower rates of proliferation and migration in vitro.

Accumulating evidence based on cell culture and on animal and human studies suggests a possible involvement of PSCs in diabetes. It has been reported that both hyperglycaemia and hyperinsulinemia increase activation (as evidenced by elevated α-SMA expression and increased synthesis of collagen and fibronectin) [83,84] and proliferation of PSCs [85]. In vitro, a co-culture study of rat PSCs with the rat β cell line, RIN-5F showed increased RIN-5F cell apoptosis secondary to caspase activation and depolarisation of mitochondria and inhibition of insulin secretion, suggesting a role for PSCs in inducing hyperglycaemia [86]. Similarly, co-culture of mouse PSCs with islets led to the activation of islet caspase 3 and impaired proliferation of β cells (TK6), although in the short incubation time of this study, insulin release did not appear to be affected. Notably, it has also been shown that PSC-mediated inhibition of insulin secretion and the resulting hyperglycaemia can in turn provoke further PSC activation [86].

Animal models of diabetes have shown activated PSCs around the islets and islet fibrosis with concomitant impaired glucose tolerance in Otsuka Long-Evans Tokushima Fatty (OLETF) [87] and Goto-Kakizaki (GK) rats [88]. It is interesting to note that islet fibrosis has also been found in diabetes patients who have undergone partial or total pancreatectomy for PDAC [89,90,91]. Several pathways, including the activation of the renin-angiotensin system [83,92,93], extracellular signal-regulated kinase (ERK) pathway [85] as well as increased oxidative stress [94] in PSCs have been postulated as possible mechanisms of diabetes-induced PSC activation in the islets. Surprisingly, Lee et al. [89] reported that the inhibition of PSCs with the anti-fibrotic agent pirfenidone lessened PSC activation and islet fibrosis in pancreas of OLETF rats, as measured by α-SMA and picrosirius red positive staining, but showed no effects on glucose tolerance and β cell function in vivo. While the literature to date supports the concept that ISCs may play a role in inhibiting β cell function in diabetes, most likely via mediators that affect β cells in a paracrine manner, little is known about the possible effects of PSCs on distant targets relevant to diabetes, namely peripheral cells, such as hepatocytes, adipocytes and skeletal myocytes, that regulate glucose homeostasis via insulin-mediated utilisation of glucose.

As noted earlier, clinical observations indicate that pancreatic cancer-related diabetes (PCRD) differs from type 2 diabetes, in that it may present as a harbinger of asymptomatic PDAC. PCRD is most likely a paraneoplastic phenomenon resulting from factors released by the cancer that negatively impact islet cell function as well as peripheral insulin sensitivity [8]. In this regard, the focus to date has been mainly on secretions from cancer cells themselves, with little attention paid to the stromal cells, namely PSCs that interact closely with cancer cells to facilitate cancer progression.

**Figure 3:**
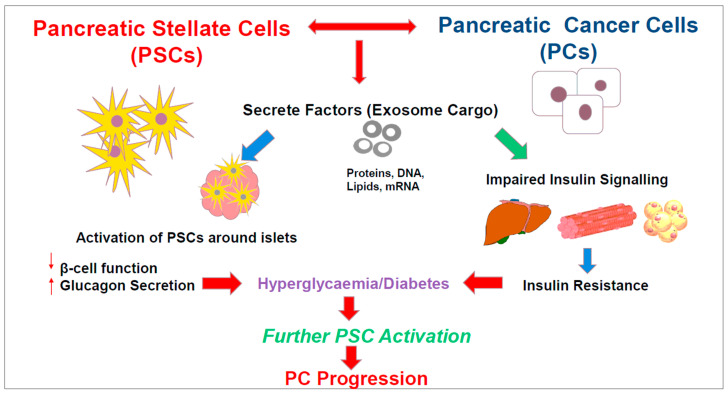
The figure depicts the concept that pancreatic stellate cells and cancer cells (including PanINs) secrete factors that are carried by exosomes to (i) activate PSCs in and around islets causing islet cell dysfunction and (ii) impair the insulin signalling pathway in hepatocytes, adipocytes and skeletal myocytes leading to insulin resistance. These effects cause hyperglycaemia/diabetes in the early asymptomatic stage of PDAC. Hyperglycaemia can lead to further PSC activation, which in turn drives accelerated PDAC progression.

In view of the discussion above regarding the role of PSCs in diabetes and their critical role in cancer progression, we are now proposing a role for these cells in PCRD. We speculate that mediators from PSCs and PanINs and cancer cells can have a deleterious effect on islet cell function and peripheral insulin resistance. The resulting hyperglycaemia further activates PSCs, which in turn drive cancer cell growth and metastasis. Since PSCs, PanINs, islets and the cells that are involved in glucose homeostasis (hepatocytes, myocytes and adipocytes) are located remotely from each other, extracellular vesicles, such as exosomes, may play a part in carrying possible mediators from the cancer/stromal tissue to islets and peripheral cells in PCRD, as illustrated in Figure 3. Analysis of the exosomal cargo using ‘multiomics’ approaches and identification of biomarkers will potentially aid in the early detection of PDAC in patients with new-onset diabetes and open up new possibilities in therapy.

## 6. Exosomes and Their Role in Pancreatic Ductal Adenocarcinoma

**Figure 4:**
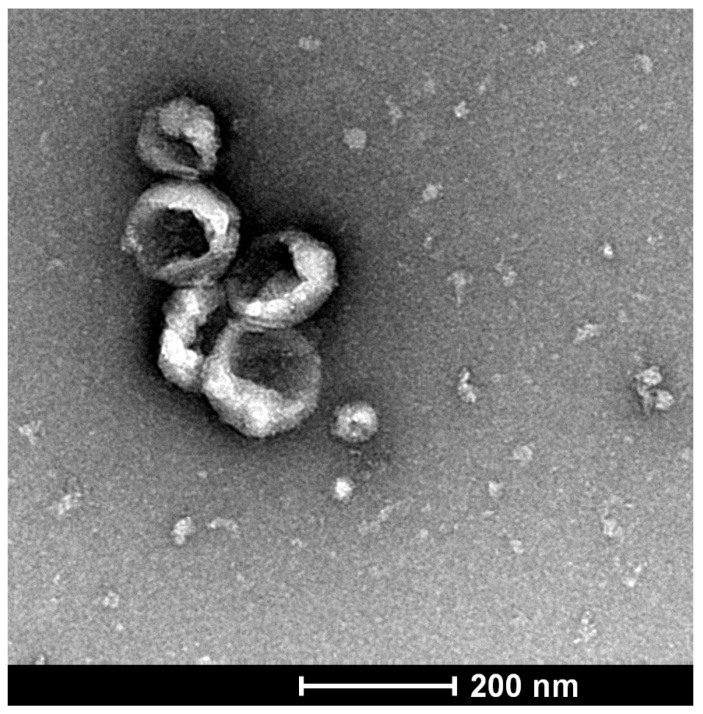
The figure depicts a cluster of exosomes isolated from pancreatic stellate cells from C57BL/6 mice. The exosomes are within their expected size range of 40–160 nm and confirm the typical cup-shaped morphology (original figure from the authors’ work).

Exosomes are gaining increasing recognition regarding their role in cell-cell communication, carrying of cargo and tumorigenesis. Exosomes are small (40–160 nm) extracellular vesicles formed from late endosomes, which exhibit a typical cup-shaped morphology, as seen by the transmission electron microscopy (Figure 4).

**Figure 5:**
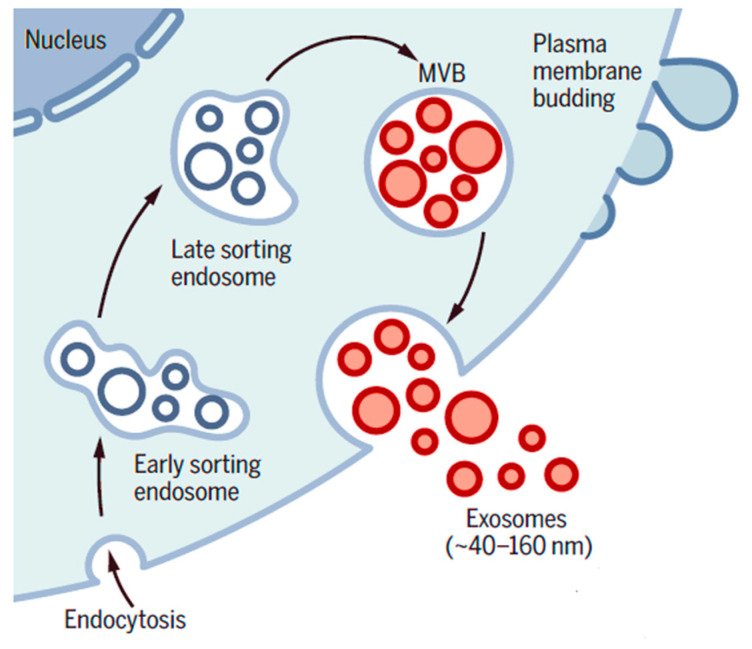
The figure depicts the genesis of exosomes from the endosomal pathway by the formation of early sorting endosomes (ESEs), late sorting endosomes (LSEs) and ultimately multi-vesicular bodies (MVBs), which contain intraluminal vesicles (ILVs). When MVBs fuse with the plasma membrane, exosomes are released (size range ~40 to 160 nm). Figure adapted from Kalluri et al. [24] with permission.

Endosomes are formed when the plasma membrane invaginates (folds inward) and encloses cytoplasmic RNAs and functional proteins, a process initiated by a ceramide-triggered mechanism. Early endosomes mature into late endosomes containing intraluminal vesicles and form multivesicular bodies (Figure 5).

Secreted exosomes are taken up by the recipient cells through various mechanisms, such as macropinocytosis, phagocytosis, clathrin-mediated endocytosis, caveolin-dependent endocytosis and plasma membrane fusion [95]. Once internalised, exosome may either fuse with the early sorting ESEs, fall apart and release their content or undergo lysosomal degradation [24]. Inside the recipient cells, exosomes may play a wide range of roles, including cell transformation by inducing mutations [96], reprogramming [97], exerting pro-inflammatory effects [98], cytokine release [99] and pro-tumorigenic effects [100].

Exosomes are secreted by most cells and are known to bear the signature of the original cell type. They are involved in cell-cell communication and carriage of intra-cellular material, such as protein, lipid, DNA and different RNA species, between cells. Interestingly, given the enclosed environment, the cargo is protected from enzymatic digestion and neutralisation. Although the exact role of exosomes in health is largely unknown, their roles, particularly that of cancer cell-derived exosomes, are increasingly being studied with respect to tumorigenesis, the tumour microenvironment and metastasis [101]. It is believed that cancer patients have more circulating exosomes than healthy subjects [102]; thus, they may serve as potential diagnostic and prognostic markers as well as novel drug delivery agents in many types of cancer.

Recent research has implicated possible roles of exosomes in progression of PDAC, including induction of cell proliferation, invasion, metastasis and chemoresistance [103,104]. Exosomes have garnered these capabilities owing to the presence of specific proteins, microRNAs, chemokines and other components in their cargo. For example, Li et al. [105] demonstrated that pancreatic tumour-secreted exosomes contain miR-222, which induces adjacent cancer cell proliferation. Another study by Fu et al. [106] revealed that downregulation of exosomal miR-98-5p increases PDAC cell proliferation by downregulating Mitogen-Activated Protein Kinase Kinase Kinase Kinase 4 (MAP4K4). Furthermore, exosomes may facilitate invasion and metastasis by modulating the tumour microenvironment, inducing hypoxia and inflammatory cell recruitment [107,108,109]. The involvement of PDAC cell-derived exosomes in pre-metastatic niche formation in the liver has been reported by Costa-Silva et al. [110] in a mouse model of PDAC; the authors reported that the observed effect was likely mediated via the exosomal macrophage inhibitory factor (MIF), the levels of which were found to be increased in the plasma exosomes isolated from tumour bearing mice. Accumulating evidence suggests that PDAC cell-derived exosomes express factors like Snail, and Snail target mRNAs, such as mRNA-146a that are associated with drug-resistance, leading to a reduced response to chemotherapy [111].

With regard to PSC-derived exosomal cargo, several studies examining the miRNA signature in the cargo using miRNA microarray analysis have postulated that miRNAs play a pivotal role in PSC–cancer cell interactions and cancer progression. For example, studies have reported that PSCs activated by PDAC cell-derived exosomes secrete exosomes containing miR-21 that can further stimulate cancer cell proliferation and EMT as well as stromal cell proliferation. In addition, miR-21 facilitates PDAC cell migration and EMT possibly via the Ras/ERK pathway [112,113]. Similarly, miR-451a in PSC exosomes has been shown to facilitate PDAC proliferation and metastasis [114]. A study by Li et al. [115] reported that miR-5703 is highly expressed in PSC-exosomes and promotes PDAC cell proliferation by downregulating tumour suppressor, CKLF-like MARVEL Transmembrane Domain containing Protein 4 (CMTM4). This study further implicates the miR5703-CMTM4-PAK4-PI3k/Akt nexus as a potential therapeutic and prognostic target for PDAC. Most recently, Cao et al. [116] have reported that hypoxia-induced overexpression of miR-4465 and miR-616-3p in PSC exosomes foster PDAC progression and metastasis by downregulating the Phosphate And Tensin Homologue (PTEN/AKT) pathway. Although these studies indicate the role of PSC- and PDAC-derived exosomal miRNAs in tumour progression, the role of cancer cell- and/or PSC-derived exosomal miRNAs on islet cell function and/or peripheral insulin signalling has not been reported, leaving a substantial gap in our knowledge in this field of research.

Several studies have investigated the possibility of utilising exosomes and their cargo as novel biomarkers for early detection of PDAC. One such study has explored Glypican 1 (GPC-1), a heparan sulphate proteoglycan present on the cell surface, which is reported to be enriched in PDAC cell-derived exosomes [117]. Circulating GPC-1 positive exosomes were detected in PDAC patients but not in healthy controls. Furthermore, the levels of GPC-1 expressing exosomes were closely related to the tumour burden and the survival of patients before and after tumour resection [117]. In contrast, Emmanouilidi et al. [104] reported that GPC-1 was expressed in both PDAC cells and non-malignant pancreatic epithelial cells without showing a significant difference in expression between the groups. Moreover, Castillo et al. [118] failed to detect GPC-1 in PDAC cell-derived exosomes implicating the heterogeneity and complexity of biomarker discovery using a single marker. However, several exosomal microRNAs, including miR-17-5p, miR-21, miR-155, miR-10b, miR-550, miR-196a, miR-5703 and miR-124b [115,119,120,121], have been suggested as useful diagnostic markers of PDAC.

In addition to exosomal miRNAs, several long non-coding RNAs (lncRNAs) [122,123], other subspecies of RNAs [124], glycoproteins, such as integrins [110], and surface and exosome cargo proteins [118] have been reported to be involved in PDAC. These may prove to be potentially useful diagnostic, therapeutic or prognostic biomarkers of PDAC. However, the use of such markers in clinical settings needs further exploration and validation.

## 7. Role of Exosomes in Pancreatic Cancer-Related Diabetes

A small number of recent studies have investigated the effects of PDAC cell-derived exosomes on a variety of tissues, including islets [26], adipose tissue [27], peripheral blood mononuclear cells [125] and skeletal muscle [126]. In an editorial on one of the studies [26], PCRD was called an “exosomopathy” [127], in recognition of the significant role exosomes might be playing in causing the disease.

Javeed et al. [26] reported that adrenomedullin (AM), a 52 kDa peptide carried by PDAC cell-derived exosomes may be responsible for reduced insulin secretion by β cells, ostensibly through adrenomedullin-induced ER stress and a defective unfolded protein response. Cancer-cell derived exosomes containing adrenomedullin have also been shown to increase lipolysis in both mouse and human adipocytes, supporting adrenomedullin as a possible mediator of PDAC-associated metabolic alterations [27]. Interestingly, data from a clinical study by Aggarwal et al. [25] indicate that serum AM levels are higher in PDAC patients with diabetes than in PDAC patients without diabetes, with both being higher than healthy controls. These findings suggest that both cancer and diabetes may be independently associated with increased serum AM. This notion is further supported by studies referred to in a review article by Wong et al. [128] reporting higher AM levels in type 2 diabetes patients, particularly in patients with complications such as diabetic retinopathy and nephropathy.

Interestingly, cancer-associated PSC-derived exosomes have also demonstrated the presence of adrenomedullin. Incubation of PSC derived-exosomes with mouse adipocytes (3T3L1) and human adipocytes resulted in increased lipolysis in these cells as assessed by glycerol release assay, indicating a possible role of adrenomedullin in obesity, but this study did not address the role of cancer-associated PSC-exosomes in PCRD [129]. Wang et al. [126] investigated the effects of exosomes derived from a mouse PDAC cell line, KPC, on insulin resistance in mouse skeletal myocytes (C2C12 cells) and observed that KPC exosomes inhibit glucose uptake in these cells. In this study, miRNA microarray and Kyoto Encyclopaedia of Genes and Genome (KEGG) analysis indicated that these effects were mediated via miR-450b-3p and miR-151-3p carried by cancer cell-exosomes, acting through the PI3K/Akt/FoxO1 pathway [126]. Furthermore, this study also demonstrated significant inhibition of the glucose transporter, GLUT4 in myocytes upon exposure to KPC exosomes. Downregulation of FoxO1 reversed the above effects of KPC exosomes.

As is clear from the above, most studies on exosome cargo to date have been limited to identifying microRNA signatures that may play a role in PCRD. However, the other components of exosomal cargo, including proteins, lipids, mRNA, long non-coding RNA and other types of RNAs, remain largely uncharacterised. Given current technological advances, a multi-omics (proteomic, lipidomic, metabolomic, genomic) approach to the study of cancer cell- and PSC-derived exosomes is eminently feasible and needs to be undertaken. Such characterisation would also aid the analysis of exosomes in liquid biopsy samples, such as plasma and serum, since exosomes in the circulation carry the signature of their parent (source) cells. Thus, once the exosomal cargo of cancer cell- and PSC-derived exosomes is characterised, it is possible that these specific signatures can be identified in circulating exosomes isolated from the plasma or serum of patients—an exciting prospect that will bring us a step closer to early detection of pancreatic cancer.

## 8. Summary

As discussed above, early detection of PDAC is vital for improved patient outcomes. The observation that a significant proportion of patients with PDAC report new onset diabetes, provides an opportunity to filter a high-risk group of patients with developing PDAC. The emerging role of exosomes in the pathogenesis of PDAC and evidence (albeit limited) of their potential use as diagnostic markers and novel therapeutic agents are fascinating. Given the increasingly well-recognised role of the stroma in cancer progression, it would be unwise to ignore the potential contribution of stromal factors in PCRD. In particular, it would be important to assess the influence of the key cells responsible for producing the stroma of PDAC, namely pancreatic stellate cells. Interestingly, our recent preliminary studies have shown that exosomes derived from co-cultures of mouse PSCs and cancer cells cause mouse beta cell dysfunction as evidenced by significantly reduce insulin secretion [130]. However, detailed in vivo and in vitro studies of the interactions of PSCs and cancer cells in the setting of hyperglycaemia or diabetes would be required to understand the pathogenesis of PCRD and the link between PCRD and cancer progression. We believe that this is an area ripe for further research—one that may unveil novel biomarkers and therapeutic targets that will aid early detection and improved treatment of this devastating condition.

## Figures and Tables

**Table 1:** Comparison between the Pancreatic Cancer-Related Diabetes and Type 2 Diabetes Mellitus.

Type 2 Diabetes Mellitus	Pancreatic Cancer Related Diabetes (PCRD)
Not associated with a tumour	Resolution of Diabetes upon surgical resection of the pancreatic tumour
Commonly associated with weight gain	Associated weight loss before the onset of diabetes
Diabetes often improves alongside weight loss	Glycaemic control worsens alongside weight loss

## Data Availability

Data on exosome figure is available. No other original data is in this manuscript.
